# Measuring arterial oxygen saturation from an intraosseous photoplethysmographic signal derived from the sternum

**DOI:** 10.1007/s10877-019-00289-w

**Published:** 2019-02-25

**Authors:** Erik Näslund, Lars-Göran Lindberg, Iréne Lund, Lui Näslund-Koch, Agneta Larsson, Robert Frithiof

**Affiliations:** 1 Department of Surgical Sciences, Section of Anaesthesia & Intensive Care, Uppsala University, Uppsala, Sweden; 2 Centre for Research & Development, Uppsala University/Region Gävleborg, Gävle, Sweden; 3 Department of Biomedical Engineering, Linköping University, Linköping, Sweden; 4 Department of Physiology & Pharmacology, Karolinska Institutet, Stockholm, Sweden; 5 Department of Medicine, Bornholm Hospital, Rønne, Denmark; 6 Department of Anaesthesia, Gävle Hospital, 801 87 Gävle, Sweden

**Keywords:** Photoplethysmography (PPG), Bone tissue, Monitoring, Respiration, Hypoxia, Human

## Abstract

Photoplethysmography performed on the peripheral extremities or the earlobes cannot always provide sufficiently rapid and accurate calculation of arterial oxygen saturation. The purpose of this study was to evaluate a novel photoplethysmography prototype to be fixed over the sternum. Our hypotheses were that arterial oxygen saturation can be determined from an intraosseous photoplethysmography signal from the sternum and that such monitoring detects hypoxemia faster than pulse oximetry at standard sites. Sixteen healthy male volunteers were subjected to incremental hypoxemia using different gas mixtures with decreasing oxygen content. The sternal probe was calibrated using arterial haemoglobin CO-oximetry (S_a_O_2_%). Sternal probe readings (S_RH_O_2_%) were then compared to S_a_O_2_% at various degrees of hypoxia. The time to detect hypoxemia was compared to measurements from standard finger and ear pulse oximeters. A significant association from individual regression between S_RH_O_2_% and S_a_O_2_% was found (r^2^ 0.97), Spearman R ranged between 0.71 and 0.92 for the different inhaled gas mixtures. Limits of agreement according to Bland–Altman plots had a increased interval with decreasing arterial oxygen saturation. The sternal probe detected hypoxemia 28.7 s faster than a finger probe (95% CI 20.0-37.4 s, p < 0.001) and 6.6 s faster than an ear probe (95% CI 5.3–8.7 s, p < 0.001). In an experimental setting, arterial oxygen saturation could be determined using the photoplethysmography signal obtained from sternal blood flow after calibration with CO-oximetry. This method detected hypoxemia significantly faster than pulse oximetry performed on the finger or the ear.

## Introduction

Measurement of arterial oxygen saturation with a multi-wavelength pulse oximeter is based on photoplethysmography (PPG). The PPG electrical signal is constituted by an alternating current (AC) caused by arterial pulsations and a direct current (DC) representing the baseline or the non-pulsatile component of the PPG-signal. The latter is alleged to reflect the total illuminated blood volume [1, 2]. Traditional pulse oximeters use both the AC- and DC-components to discriminate arterial from venous blood and to calculate arterial oxygen saturation [3].

A pulse oximeter is most commonly placed on the peripheral extremities. Low perfusion states, for instance during peripheral vasoconstriction or hypothermia, may interfere with the estimation of arterial oxygen saturation from peripheral sites by reducing PPG-signal strength [1, 4, 5].

Also, detection of a decline in core arterial oxygen saturation is delayed if the measurement is performed peripherally compared to a central location [1, 6–9]. Given these limitations of peripheral pulse oximeters, a theoretically more appealing solution would be to assess core arterial oxygen saturation in centrally located vessels not equally affected by peripheral vasoconstriction. The purpose of this study was to evaluate the measurements of intraosseous oxygen saturation from the sternum utilising only the DC-component of the PPG-signal from a novel PPG-prototype.

We hypothesised the following:

- Arterial oxygen saturation can be estimated with PPG-recordings obtained from sternal blood flow once calibrated with arterial haemoglobin CO-oximetry.
- Hypoxemia, in this study defined as the lowest observed oxygen saturation, will be detected faster when arterial oxygen saturation is monitored on the sternum as compared to other standard sites for pulse oximetry.

## Methods

The study was performed in collaboration with the Swedish Armed Forces and took place at the Diving and Naval Medicine Center (SwAF DNC) in Karlskrona, Sweden.

A Regional Ethical Review Board approval (EPN, Stockholm application number 2014/2017-31/4) was obtained before participants associated with SwAF DNC were recruited by advertising. Potential candidates were interviewed about their current and past medical status by a physician to determine their suitability to participate in the study. Sixteen healthy men were enrolled into the study (Mean (SD)–Age (y) 39.9 (11.1); Weight (kg) 89.3 (9.5); Height (m) 1.85 (0.08); BMI (kg m^− 2^) 25.9 (2.0)). Informed consent was obtained from all individual participants included in the study.

Criteria for inclusion were voluntariness, healthiness and good physical condition. Exclusion criteria included respiratory tract infection, cardiopulmonary disease, smoking, age < 18 years and occurrence of previous fracture/surgery of the sternum. Participants could at any time choose to discontinue the trial.

The experiment was interrupted if a participant reached an arterial oxygen saturation below 60% measured either by arterial haemoglobin CO-oximetry or by reference pulse oximetry. Other criteria for discontinuing the experiment were a lack of ability among the participants to respond to repeated communication using hand signs or if someone should show signs of clinical respiratory distress.

### The sternal probe

**Fig. 1:**
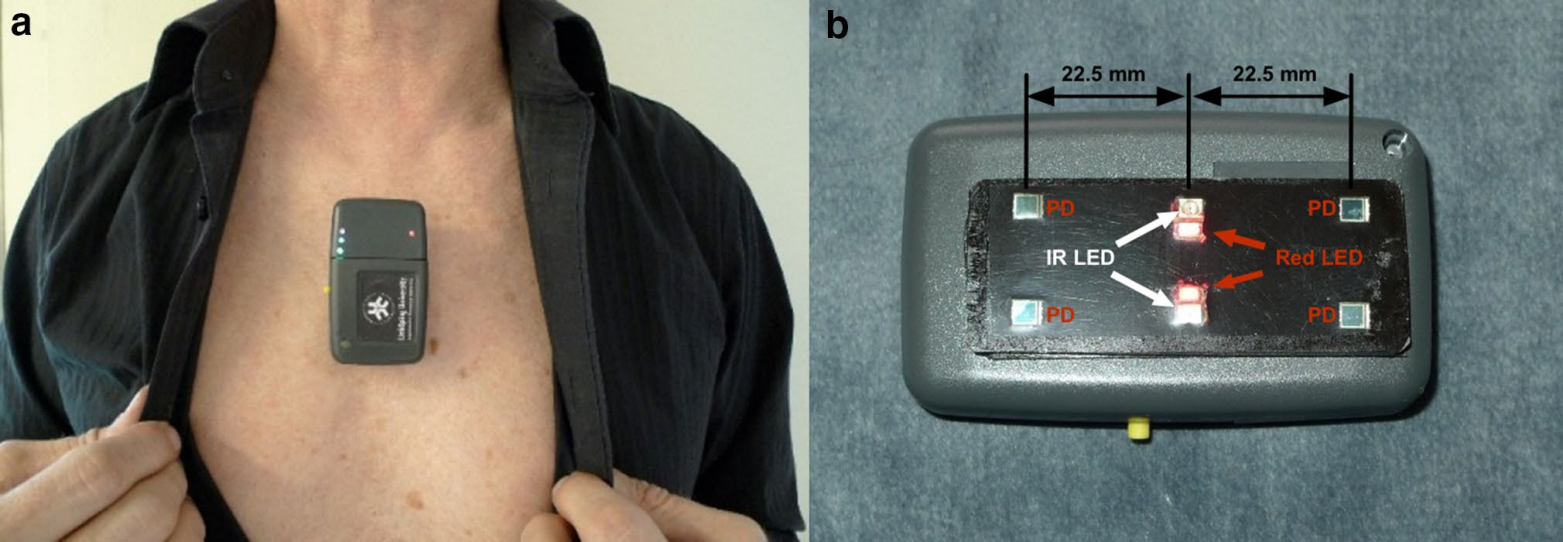
The sternum probe **a** positioned over the sternal corpus. **b** The sensor plate with four photodetectors (PD) and two infrared LED (810 nm) and two red LED (660 nm). The centre-to-centre distance between LED and PD is 22.5 mm

The novel device used is a research prototype sternal probe developed by the Department of Biomedical Engineering, Linköping University, Sweden and is named RespiHeart (RH). It is attached to the skin on the sternum using a medical grade double adhesive tape and works by reflectance mode PPG. Two infrared and two red light emitting diodes (LED) together with four photodetectors (PD) in a dual lining configuration is used to cover blood flow bilaterally in the bone (Fig. 1). The components are embedded in a 25 × 62 mm black Polyurethane sensor plate. Based on previous studies [2, 10, 11] the centre-to-centre distance between LED and PD was chosen to be 22.5 mm to assure an adequate intraosseous depth of PPG-measurement. The double lining configuration of LED and PD enables a relatively large volume of blood to be analysed which entails a high signal to noise ratio (SNR) and subsequently a PPG-signal of high quality.

Wavelengths of the LED light sources are 660 nm (red), which is oxygen sensitive, and 810 nm (infrared). The latter is close to the isosbestic point and acts as a reference during oxygen saturation measurements. Arterial oxygen saturation derived from the probe is determined according to the equation:

1 $${{\text{S}}_{{\text{RH}}}}{{\text{O}}_2}\% ={{\text{f}}_{{\text{RH}}}} \times {\text{DC}}\left( {810{\text{ nm}}} \right)/{\text{DC}}\left( {660{\text{ nm}}} \right)={{\text{f}}_{{\text{RH}}}} \times {{\text{R}}_{{\text{DC}}}}$$

f_RH_ is a functionality constant, and DC is the light registered from the DC-component of the PPG-signal.

Readings from the sternal probe are transferred via Bluetooth^Ⓡ^ communication to a signal acquisition program on a personal computer (Windows 7 Professional, Microsoft, Redmond, Washington, USA) with a sampling rate of 100 Hz.

### Preparations of participants

The participants were placed in a comfortable supine position. In each participant, a 20 G indwelling arterial cannula was inserted into the right radial artery after infiltrative local anaesthesia with Lidocaine 1%. The sternal probe was attached to the cranial part of the sternal corpus, below the manubrium. A dark piece of cloth covered the sternal probe to reduce the inflow of ambient light. The intensity of emitted light from the probe and amplification of reflected light was adjusted using gain to achieve an adequate voltage/signal in the signal acquisition program.

Electrocardiogram (ECG), invasive arterial blood pressure and pulse rate were continuously monitored with Corpuls^3^ (GS Elektromed, Geräte G Stemple GmbH). Oxygen saturation was continuously monitored with pulse oximetry with sensors placed over the left index finger (RD Set, Masimo, Irvine, CA) and left earlobe (E1, Radical7, Masimo, Irvine, CA). Since no digital data output was available from Corpuls^3^ and the finger pulse oximeter, the entire experiment was filmed.

### Inducing hypoxia

The participants breathed gas mixtures with decreasing oxygen, O_2,_ content, from different pre-filled gas cylinders. The hypoxic gas mixtures were produced by diluting room air with nitrogen to achieve an O_2_ content of 18%, 15%, 12%, 9% and 6%, respectively. The exact O_2_ content in each cylinder was checked by mass spectroscopy (AMIS 2000, Innovision, Glamsbjerg, Denmark) before the commencement of the study. Gases were delivered by a mouthpiece on a regulator connected via a T-piece to two gas cylinders. Changes in breathing gas were made by shifting the high flow from the separate cylinders. Direct end-tidal O_2_ and CO_2_ were measured via a sampling line in the mouthpiece and analysed by Servopro 1440 (Servomex, Crowborough, UK).

### Experimental design

**Fig. 2:**
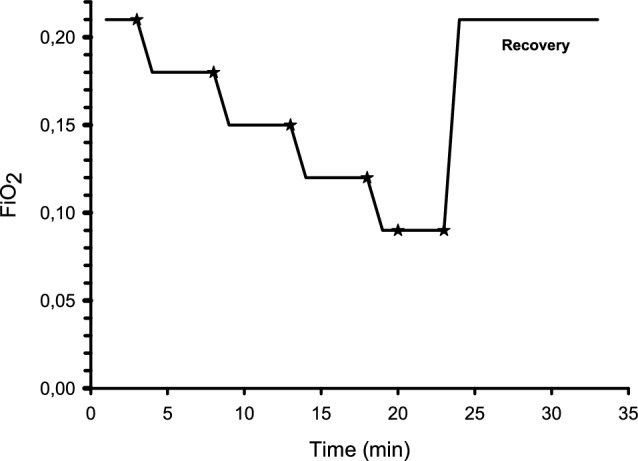
Experimental time course illustrating reductions in FiO_2_, i.e. when breathing gases were changed. Stars indicate when arterial blood was sampled for arterial haemoglobin CO-oximetry. For each occasion, two samples were obtained. The experiment ended with a recovery period of 10 min, and then the participant was breathing room air

The participants started breathing 21% O_2_ (room air). Every 5 min the oxygen content was successively reduced in steps of 3% units down to 9%. Two arterial blood samples were collected at the end of each step. A time clock was used to note the exact time when the samples were collected. For the 9% level, two additional samples were collected after 2 min (Further referred to as 9a and 9b, respectively). All arterial blood samples were analysed with CO-oximetry (ABL90 Flex analyzer, Radiometer Medical ApS, Denmark). Participants who did not reach a desired arterial oxygen saturation level were either asked to breathe the 9% gas mixture for an additional minute or to shift to a gas cylinder with an oxygen content of 6%, with either one or two samplings (denoted 6a respectively 6b). The experiment ended with a recovery period of 10 min during which the participant breathed room air (Fig. 2).

### Data handling

Collected DC (810/660 nm) raw signal from the probe was analysed in Matlab (R2014a, Mathworks, Massachusetts, USA). The 100 Hz, PPG DC-raw signal, was transformed using a rolling average to 1 Hz. Any prior adjustment of gain was corrected before further analysis.

The finger and the ear sensor had a rolling average of 8 and 2 s respectively, and was updated every second. All signals were processed post hoc to have the same rolling average of 8 s.

**Fig. 3:**
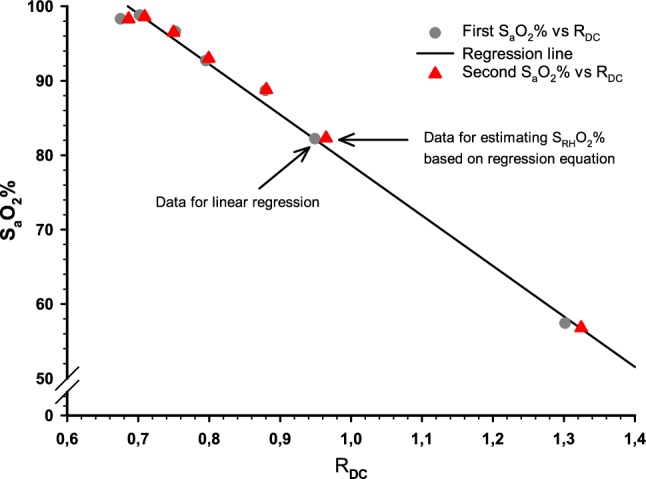
Scatterplot of one selected participant illustrating the data handling. The plot illustrates the first and second of the paired collected arterial samples (S_a_O_2_%) against the measured DC-quotient (R_DC_) from the sternal probe

For each individual, the relationship between the quotient of the corrected DC_IR_/DC_Red_ (R_DC_) and the first of the paired collected arterial samples was estimated separately, using simple linear regression analysis. The quotient chosen for analysis was the one corresponding to the time when the arterial blood sample was collected. Using these fitted regression models, each corresponding individual sternum probe assessment of arterial oxygen saturation (S_RH_O_2_ %) was estimated from the second of the paired arterial samples (Fig. 3).

### Statistics

As no similar studies using RespiHeart have been undertaken previously, an a priori power calculation could not be performed. Normally distributed variables are summarised as mean (SD) and non-normally distributed values as median (IQR[range]).

The variables arterial oxygen saturation (S_a_O_2_%) and the corresponding value of S_RH_O_2_% were not normally distributed according to a normal probability plot together with the Shapiro–Wilks test. For that reason, non-parametric statistics were used to assess the relationship between S_a_O_2_% and S_RH_O_2_%.

Analysis of S_a_O_2_% and S_RH_O_2_% were done separately for each of the different gas levels, and their association was tested with Spearman Rank Order correlation. The agreement between S_a_O_2_% and S_RH_O_2_% for the different gas mixtures was evaluated using Bland–Altman plots [12, 13].

**Fig. 4:**
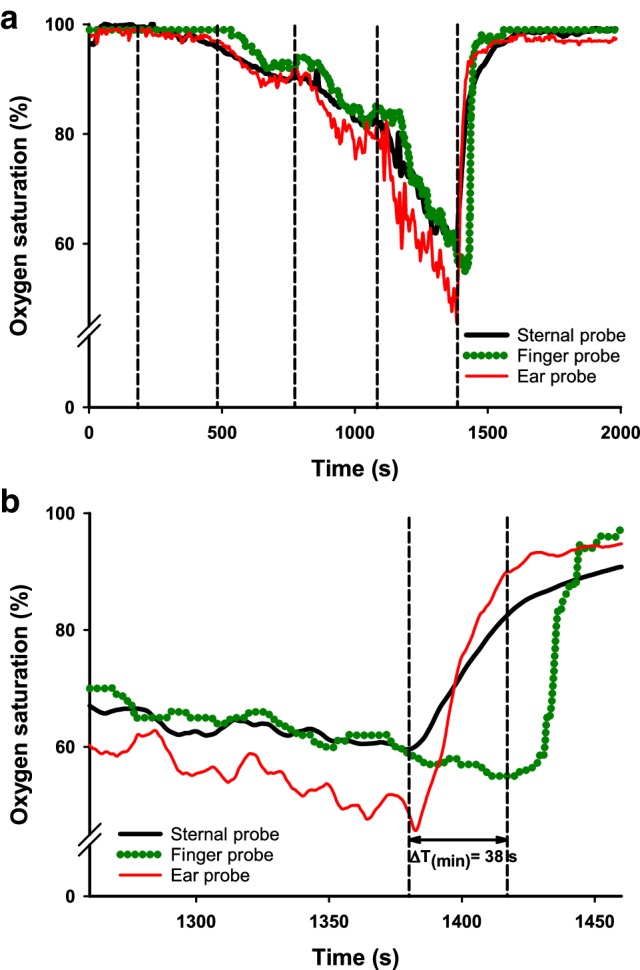
Line plot, of one selected participant with a typical response pattern (**a**) illustrating the different modalities for continuously measuring arterial oxygen saturation; sternal probe, finger probe and ear probe. Vertical lines denote the changes of inhaled oxygen content. The last change is to room air. **b** Is a magnification of 4a where the lowest observed oxygen saturation is noted. ΔT(min) is the time difference (seconds) between the sternal probe and the finger probe

Line plots of continuously measured oxygen saturation against time were used for the different measurement modalities (sternal probe, finger probe and ear probe) to identify the lowest observed oxygen saturation (Fig. 4). Data of continuously measured arterial oxygen saturation followed a normal distribution, according to a normal probability plot with Shapiro–Wilks test. Statistical analysis of time differences between the various modalities was performed with one-way repeated measurement ANOVA.

A two-sided p-value < 0.05 was considered to be statistically significant.

All statistical calculations were performed with Statistica 13 (Dell Statistica, Tulsa, OK, USA) and all graphs were created with SigmaPlot 13 (Systat Software Inc, San Jose, CA, USA).

## Results

S_a_O_2_%-measurements from two participants were excluded due to blood clotting during arterial haemoglobin CO-oximetry and failed synchronisation between arterial blood sampling and sternal probe readings, respectively. All participants were included in the time-to-minimum saturation analysis.

During the trial, no adverse events were reported from any of the participants, and they could all complete the study protocol.

**Fig. 5:**
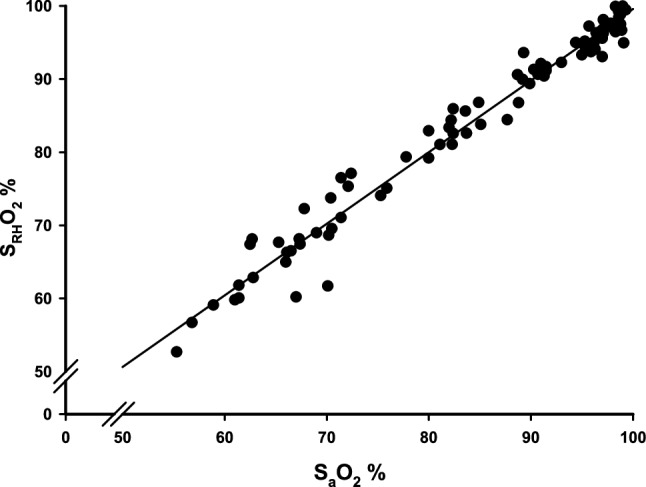
A scatterplot showing the association between measured arterial oxygen saturation from arterial haemoglobin CO-oximetry (S_a_O_2_%) and probe derived oxygen saturation (S_RH_O_2_%) calculated via the quotient R_DC_ obtained from individual measurements (n = 14). R^2^ = 0.974

S_RH_O_2_% was calculated from the individual regressions. The coefficient of determination for the relationship between S_a_O_2_% and S_RH_O_2_% was 0.974, p < 0.001 (Fig. 5). Spearman rank order correlations demonstrated a significant association between arterial haemoglobin CO-oximetry and the sternal probe readings for every gas level tested, range 0.712–0.924 (Table 1). Data are not shown for the 6% oxygen level due to too few observations. A total of 184 arterial blood samples were collected, 12 were derived from the 6% oxygen level. Also, these 12 samples originated from only 4 out of the 14 subjects. Table 2 shows a summary of mean (SD) values for all S_a_O_2_% and the corresponding S_RH_O_2_%, acquired at each inhaled gas mixture.

**Table 1:** Association between S_a_O_2_% and S_RH_O_2_% tested with Spearman rank order correlations between the various gas levels (n = 14)

Inhaled oxygen content (%)^a^	Spearman R	p-value
21	0.712	0.00431
18	0.773	0.00118
15	0.898	< 0.001
12	0.850	< 0.001
9a	0.916	< 0.001
9b	0.924	< 0.001

S_a_O_2_% Measured arterial oxygen saturation from CO-oximetry

S_RH_O_2_% Arterial oxygen saturation obtained from the sternal probe readings

^a^9a, 9b% denotes the two separate samplings of arterial blood at the 9% inhaled oxygen content

**Table 2:** Summary of S_a_O_2_% and S_RH_O_2_% values acquired for each inhaled gas level

Inhaled oxygen content (%)^a^	S_a_O_2_% Mean (SD)	S_RH_O_2_% Mean (SD)	n
21	98.1 (1.0)	97.1 (2.1)	14
18	96.9 (1.4)	96.5 (1.9)	14
15	93.3 (3.3)	93.3 (3.1)	14
12	85.4 (6.9)	86.3 (6.0)	14
9a	75.8 (8.1)	76.9 (8.6)	14
9b	71.4 (10.8)	71.1 (10.8)	14
6a	64.9 (5.4)	66.3 (6.4)	4
6b	56.0; 65.8	54; 67	2

S_a_O_2_% Measured arterial oxygen saturation from CO-oximetry

S_RH_O_2_% Arterial oxygen saturation obtained from the sternal probe readings

*SD* standard deviation

^a^a-b% denotes the two separate samplings of arterial blood at the 9% respectively 6% inhaled oxygen content. For 6b%, the individual values are presented

**Fig. 6:**
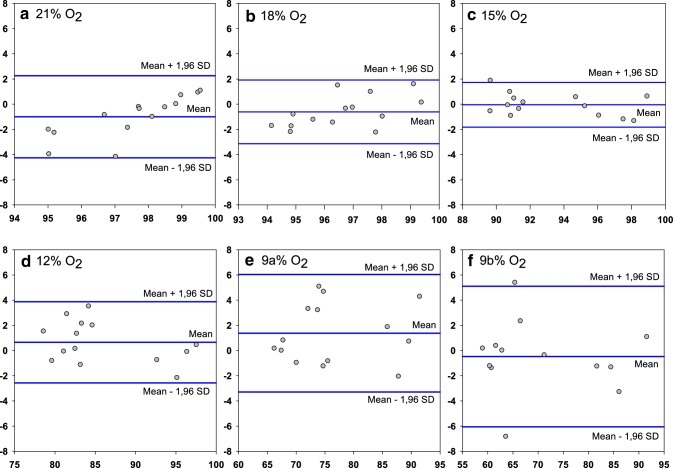
Bland–Altman plots comparing results from measured arterial oxygen saturation (S_a_O_2_%) and sternal probe readings (S_RH_O_2_%) obtained breathing the different inhaled gas mixtures (n = 14). Data for each plot is presented as mean difference (SD), lower limit of agreement (− 1.96 SD) and higher limit of agreement (+ 1.96 SD). **a** − 0.997 (1.66), − 4.26, 2.26, **b** − 0.609 (1.29), − 3.13, 1.92, **c** − 0.0437 (0.91), − 1.82, 1.73, **d** 0.656 (1.64), − 2.57, 3.88, **e** 1.37 (2.38), − 3.29, 6.04, **f** − 0.471 (2.85), − 6.05, 5.11

The variances of collected S_a_O_2_% and S_RH_O_2_% followed each other but were unevenly distributed for the different gas mixtures, being the highest at the lowest inhaled oxygen level. Also, the distribution of the variances was not linear. As a consequence, we were unable to analyse the agreement between S_a_O_2_% and S_RH_O_2_% on a group level. Limits of agreement between S_a_O_2_% and S_RH_O_2_% in Bland–Altman plots are presented separately for each administered gas mixture (Fig. 6).

The limits of agreement are increased with decreasing oxygen saturation. All gas mixtures, except for the 12% and 9% O_2_-level, had a negative bias indicating that the sternum probe slightly overestimates the oxygen saturation compared to arterial haemoglobin CO-oximetry.

Figure 4a illustrates the entire course of a selected, typical experiment in one participant. In Fig. 4b the recording of the lowest observed oxygen saturation in the same experiment is visualised with higher temporal resolution. The sternal probe was on average 28.7 s (95% CI 20.0–37.4 s, p < 0.001) faster than the finger probe in detecting the lowest measured oxygen saturation. Compared to the ear probe, the sternum probe was on average 6.6 s faster (95% CI 5.3–7.8 s, p < 0.001).

## Discussion

This is the first test of the novel PPG sensor prototype (RH) in human hypoxia. The same type of sensor has previously been tested on emergency ward patients with a focus on monitoring heart and respiratory rate [14]. Our results indicate that monitoring arterial oxygen saturation from sternal blood flow using the DC-component from PPG-measurements is feasible. Furthermore, the time to detect the lowest observed oxygen saturation is significantly shorter when monitoring oxygen saturation over the sternum as compared to other standard monitoring sites using conventional methods.

The PPG-technique has previously been used for studying blood flow in superficial soft tissues, such as skin and muscle [2, 15, 16]. Recordings from deeper tissues need adjustments of light wavelengths and distances of light source and photodetectors. Accordingly, similar optical techniques such as Near Infrared Spectroscopy (NIRS), utilises long distances between the light source and the photodetector [17].

The current device, RH, also uses an increased distance between light source and detector and is therefore able to measure blood flow in deeper tissue compartments, in this case the sternum. Opposite to a probe using the same wavelengths, but with a centre-to-centre distance of 6.5 mm between LED light source and photodetector, the pulsatile RH-signal does not change when skin blood flow is increased/decreased using manual skin compression and vasodilation with Methylnicotinate, respectively (unpublished observations). This indicates that the RH-signal is not derived from skin blood flow.

Using PPG on bone tissue has attracted increasing interest. Bone tissue has been demonstrated to be optically transparent allowing PPG assessment and measurements have been performed in patella and tibia that correlates to blood flow in the same tissue [10, 11]. Furthermore, bone blood flow remains stable despite external influences such as pressure or exposure to low temperatures [18].

The sternum has a central anatomical location close to the heart and receives high and constant blood flow from several major arteries [19, 20].

The subcutaneous tissue covering the sternum is often thin, and the bone itself lacks pigment, resulting in only a modest interference of melanocytes and subcutaneous blood flow on light absorption and scattering. These factors make the sternum a suitable location for reflective PPG-measurements.

Pulsatile changes have been considered a prerequisite for pulse oximeters to calculate oxygen saturation [21]. However, since bone tissue is rigid, no pulsatile volumetric change can give rise to the AC-signal. In this study, using only the DC-components (R_DC_), arterial oxygen saturation could be correctly estimated for each individual in comparison to measurements with arterial haemoglobin CO-oximetry.

The sternal intramedullary oxygen tension likely does not equal arterial oxygen tension. In a previous publication, the intramedullary oxygen saturation in the manubrium was estimated to be 71.1% (median, IQR 69.5–72.3%) [22]. It has been proposed that oxygen consumption in bone tissue is constant due to stable metabolism [23]. This entails a linear relationship between sternal and arterial oxygen tension. Since we have shown that there is a linear association between R_DC_ and S_a_O_2_% (Fig. 5), calibration using arterial haemoglobin CO-oximetry will allow S_RH_O_2_% to give a correct estimation of S_a_O_2_%.

According to analysis using the Spearman rank order correlation (Table 1), this association was significant for all ingoing gas mixtures. The analysis of association was only performed between S_a_O_2_% and S_RH_O_2_% since both the finger probe and ear probe have already been validated by their manufacturer respectively, and our aim was not to further evaluate this.

In the current experiments, the lowest oxygen saturation was reached at the end. The sternum probe was on average 28.7 s faster than the finger probe in detecting the lowest observed oxygen saturation. Albeit of uncertain clinical importance, the sternum probe was also significantly faster than the ear probe (on average 6.6 s). This shorter response time probably reflects differences in the anatomic location of the probes.

These factors together suggest that monitoring arterial oxygen saturation in the sternum might circumvent the effects of peripheral vasoconstriction and shorten the time to detect a hypoxic event.

In this study using the sternum probe, there are some factors not compensated for, leading to inter-individual phase-shifts in the regression lines; (1) We did not control for the probe contact pressure against the skin. Anatomical and physiologic differences among the participants could affect the interaction between sensor plate and skin. The higher probe pressure against the skin the better signal acquired from the marrow, up to a certain plateau [22]. Furthermore, a possible gap between the sensor plate and the skin could have caused optical shunting of light between LED and PD. (2) The participants breathing pattern was not controlled for. The study participants tolerated hypoxemia differently, and some had a more forceful respiratory pattern with the possibility of motion artefacts. For these reasons, an aggregated calibration curve for all the participants was not calculated. The consequence of this was that the oxygen saturation could not be read instantly in percentage for each participant. However, there was a significant correlation between the sternum probe readings and oxygen saturation from arterial haemoglobin CO-oximetry (Fig. 5; Table 1) for each individual. In upcoming studies taking probe pressure and breathing variability into account, an algorithm based on an aggregated calibration curve will be used and then direct readings of individual oxygen saturation in percentage will be possible.

Constraints, in general, to consider in the present study are that only men with Caucasian origin signed up as volunteers. The spread of age was acceptable, but optimally the study population should have been more mixed with regards to gender, ethnicity and body constitution (BMI). No equipment was available to quantify the subcutaneous thickness of the skin over the sternum of the participants. Furthermore, by controlling the participants breathing pattern and the probe application pressure would have allowed us to create an aggregated calibration curve for all participants.

For each subject, the intensity of emitted light and gain was adjusted to achieve a signal with good quality. Based on the present findings, future studies will use updated software and hardware, so that an automatic adjustment of light intensity is performed to achieve a pre-defined voltage from the photodetectors. Hence, individual calibration will no longer be necessary.

Since these hypoxia experiments were performed on healthy volunteers we are so far not yet able to conclude about the clinical usefulness of the sternal probe. This was also outside the scope of the current study. Before a possible introduction in clinical practice, further investigation of the sternal physiology during various conditions should be considered.

In conclusion, the results from the current study show that it is possible to accurately determine arterial oxygen saturation changes with PPG on blood flow in the sternum using only the DC-component of the PPG-signal. The time to detect the lowest observed oxygen saturation is significantly faster using this method compared to standard monitoring sites for pulse oximetry. Continued research for corrections of inter individual-variations and the development of a population-based algorithm for arterial oxygen saturation is warranted.
